# An LC-MS Method to Quantify Rhein and Its Metabolites in Plasma: Application to a Pharmacokinetic Study in Rats

**DOI:** 10.3390/metabo15060407

**Published:** 2025-06-17

**Authors:** Nyma Siddiqui, Yuan Chen, Ting Du, Yang Wang, Charmeyce Buck, Song Gao

**Affiliations:** Department of Pharmaceutical Science, College of Pharmacy and Health Sciences, Texas Southern University, 3100 Cleburne Street, Houston, TX 77004, USA; n.siddiqui6955@student.tsu.edu (N.S.); yuan.chen@tsu.edu (Y.C.); du.ting@tsu.edu (T.D.); yang.wang@tsu.edu (Y.W.); c.buck4052@student.tsu.edu (C.B.)

**Keywords:** LC-MS, diacerein, Rhein, Rhein-glucuronides, PK

## Abstract

**Background:** Diacerein, a prodrug of Rhein, is commonly prescribed for the management of joint disorders, specifically osteoarthritis. This study aimed to develop and validate an LC-MS/MS method to quantify Rhein and its major metabolites, Rhein-G1 and Rhein-G2, in plasma samples. **Method:** An ACE C18 column was used for chromatographic separation with a mobile phase comprising ammonium acetate at a concentration of 1.0 mM and acetonitrile. Detection was achieved using a Sciex 4000 Q-Trap LC-MS/MS, operated in negative ion mode with multiple reaction monitoring (MRM). **Results:** The analytical results indicated that the lower limit of quantification (LLOQ) for Rhein and its glucuronides was 7.81 nM. Precision was consistently below 9.14%, while accuracy remained within the acceptable range of 80.1–104.2%. We also verified the method’s matrix effect recovery and stability variance, which were less than 12.60% and 10.37%, respectively. The pharmacokinetic study demonstrated that diacerein is swiftly metabolized into Rhein, and then Rhein subsequently undergoes glucuronidation, forming detectable concentrations of Rhein-G1 and Rhein-G2 in plasma. **Conclusions:** This new LC-MS/MS method proved to be both sensitive and selective, allowing for pharmacokinetic studies in rats.

## 1. Introduction

Diacerein, a pro-drug that undergoes metabolic conversion to Rhein, has been extensively used to treat inflammatory diseases such as osteoarthritis. This medication is particularly effective because of its ability to inhibit the activity of interleukin-1 beta (IL-1β) and tumor necrosis factor-alpha (TNF-α), two cytokines known for their central role in promoting inflammation and degradation of cartilage within joints. Diacerein is administered orally [1,2], making it convenient for long-term treatment. In addition to osteoarthritis, the drug has been explored for its effectiveness in managing other inflammatory diseases, such as psoriasis, due to its action on cytokine-related pathways [3]. Furthermore, diacerein exhibits broad pharmacological activities, including mitigating kidney damage caused by cisplatin, regulating blood sugar levels in diabetes mellitus, and alleviating toxin-induced liver and lung injuries [4,5,6].

After oral administration, diacerein undergoes rapid metabolic conversion in the liver and intestines to yield Rhein, the active metabolite. Rhein can be further conjugated into glucuronides by UDP-glucuronosyltransferases (UGTs) [7,8]. This conjugation enhances Rhein’s water solubility, facilitating its excretion. In rats, Rhein has been shown to be excreted in both urine and bile. Rhein-glucuronides are efficiently secreted into the gastrointestinal tract via bile, where they may undergo hydrolysis by gut microflora, releasing free Rhein that can be reabsorbed, contributing to an enterohepatic circulation [9,10]. These metabolic processes highlight the need for a comprehensive quantification of both Rhein and its glucuronides in pharmacokinetic studies.

Several analytical methods have been developed to quantify Rhein in biological samples, especially in the context of clinical or preclinical studies where Rhein is administered either as a pure compound or as part of herbal formulations [11,12]. However, to our knowledge, there has been no prior method that allows for the simultaneous quantification of Rhein and its glucuronides in plasma, especially after oral administration of the prodrug form diacerein. Therefore, this study sought to develop and validate a highly sensitive LC-MS/MS method that could quantify Rhein and its major metabolites, Rhein-G1 and Rhein-G2, simultaneously, providing a robust analytical tool for use in pharmacokinetic studies.

## 2. Materials and Methods

### 2.1. Chemicals and Materials

Diacerein and Rhein, which were used as standard compounds in this study, were purchased from Ambeed (Arlington Heights, IL, USA). The internal standard, wogonin, was obtained from Avachem Scientific (San Antonio, TX, USA). All these compounds had a purity level of 99% or higher. LC-MS grade acetonitrile and water, which are essential for achieving high sensitivity and reproducibility in mass spectrometric analysis, were procured from Thermo Scientific (Fairlawn, NJ, USA) and VWR (Radnor, PA, USA), respectively. Other reagents were used without further purification. Rhein-G1 and Rhein-G2, two important metabolites of Rhein, were synthesized using rat liver microsomes, following an established protocol [13]. The purity of the synthesized Rhein-G1 and Rhein-G2 was confirmed to be above 95% using UPLC-UV analysis. Rat liver microsomes were purchased from Xenotech Sekisui (Kansas City, KS, USA).

### 2.2. Method

#### 2.2.1. UHPLC System

Chromatographic separation was achieved using a Shimadzu UHPLC system (SCIEX, CA, USA) equipped with an ACE BEH C18 column (50 × 2.1 mm I.D., 1.7 µm; Avantor, Radnor Township, PA, USA). The mobile phase consisted of 1 mM ammonium acetate (mobile phase A) and acetonitrile (mobile phase B). The gradient elution was programmed as follows: from 0 to 0.01 min, 5% B; from 0.01 to 0.50 min, 5% B; from 0.50 to 1.50 min, 37% B; from 1.50 to 4.50 min, 98% B; and from 4.50 to 5.00 min, 98% B. The gradient was subsequently brought back to 5% B from 5.00 to 5.10 min. The flow rate was maintained at 0.4 mL/min, and the column temperature was kept at 40 °C, providing optimal separation of the compounds.

#### 2.2.2. MS System

Mass spectrometric analysis was performed on a Sciex QTrap 4000 (Marlborough, MA, USA) mass spectrometer, which was equipped with an electrospray ionization (ESI) source. The system was set to negative ion mode, utilizing multiple reaction monitoring (MRM) for detecting Rhein and its glucuronides. Both quadrupoles (Q1 and Q3) were configured to provide unit resolution, and the spray voltage was set at −4500 V. The ion source temperature was held at 500 °C, and nitrogen was used as both the nebulizer gas and turbo gas at 50 psi.

#### 2.2.3. Biosynthesis of Rhein-G1 and Rhein-G2

The biosynthesis of Rhein-G1 and Rhein-G2, the glucuronide metabolites of Rhein, was carried out using a well-established methodology that has been previously validated in our laboratory [13,14,15]. The reaction mixture consisted of 5 mM Rhein, 10 mg/mL liver microsomes, 5 mM magnesium chloride (MgCl_2_), 4.4 mM saccharolactone, 0.022 mg/mL alamethicin, and 3.5 mM UDPGA (uridine 5′-diphospho-glucuronic acid), prepared in 50 mM potassium phosphate buffer (pH 7.4) to optimize enzymatic activity. The mixture was incubated at 37 °C for six hours to allow the complete conversion of Rhein to its glucuronides. Following incubation, the reaction was halted by the addition of acetonitrile at ten times the volume of the reaction mixture. The solution was vortexed thoroughly, centrifuged, and the supernatant was collected. Solvent removal was achieved by nitrogen gas evaporation, and the residue was reconstituted in acetonitrile, forming the stock solution for subsequent analysis.

#### 2.2.4. Identification and Quantification of Rhein-G1 and Rhein-G2

The identification of Rhein-G1 and Rhein-G2 was confirmed using a combination of enzymatic hydrolysis, UV spectroscopy, and LC-MS/MS analysis. For quantification, a calibration curve for Rhein was constructed and applied to determine the concentrations of Rhein-G1 and Rhein-G2, ensuring the accurate measurement of these metabolites in the plasma.

### 2.3. Method Validation

#### 2.3.1. Calibration Curve and LLOQ (Lower Limit of Quantification)

To establish the calibration curve, standard solutions of Rhein, Rhein-G1, and Rhein-sulfate were prepared in 50% acetonitrile, with concentrations ranging from 2000.00 nM down to 3.91 nM. For plasma calibration, 20 µL of blank plasma was spiked with 20 µL of each calibration sample, followed by the addition of 400 µL of internal standard (50 nM wogonin in 50% acetonitrile). The samples were sonicated to ensure efficient drug extraction, and then centrifuged at 20,000× *g* for 15 min. The resulting supernatant was dried under nitrogen, and the residue was reconstituted in 100 µL of 50% acetonitrile for injection. Calibration curve linearity was evaluated by plotting the ratio of the analytes’ peak areas to the internal standard against the corresponding concentrations. The slope, intercept, and correlation coefficient were determined using a least-squares linear regression method with 1/x^2^ weighting. The lower limit of quantification (LLOQ) was based on a signal-to-noise ratio of 10:1, ensuring the reliable detection of low concentrations.

#### 2.3.2. Precision and Accuracy

The precision and accuracy of the method were evaluated by analyzing quality control (QC) samples at three different concentration levels. Each QC sample was injected in triplicate, both within a single day (intra-day) and across three consecutive days (inter-day). The concentrations used for these tests were 1000 nM, 500.00 nM, 15.62 nM, and 7.81 nM. Accuracy was expressed as the percentage of the nominal concentration, and precision was assessed by calculating the relative standard deviation (RSD) for repeated measurements. The results demonstrated that both accuracy and precision were within acceptable ranges according to FDA guidelines for bioanalytical method validation.

#### 2.3.3. Extraction Recovery and Matrix Effect

To assess extraction recovery, peak areas of plasma samples spiked with Rhein and its glucuronides were compared to those of analytes spiked into water. This ensured that the extraction method was efficient enough to recover the analytes from the biological matrix. The matrix effect was also investigated by comparing analyte responses in plasma extracts to those in standard solutions injected directly into the mobile phase. These evaluations followed the guidelines set forth by the U.S. Food and Drug Administration (FDA) for bioanalytical method validation [16]. The results indicated that the extraction recovery and matrix effect were within acceptable ranges (Table 1), confirming the robustness of the method.

#### 2.3.4. Stability

The stability of Rhein, Rhein-G1, and Rhein-sulfate in plasma was assessed under various storage conditions to ensure the integrity of the analytes over time. QC samples at three concentration levels were analyzed after storage at room temperature (25 °C) for six hours, at −80 °C for 60 days, and after three freeze–thaw cycles (from −80 °C to 25 °C). Stability was calculated as the percentage of the initial concentration of the analytes remaining at each time point. The analytes remained stable under all tested conditions, with concentration variations falling within the acceptable range of 80% to 120%.

### 2.4. Pharmacokinetics Study

#### 2.4.1. Animals

Pharmacokinetic studies were conducted using female Wistar rats, which were obtained from Harlan Laboratories (Indianapolis, IN, USA). The animals were housed in a controlled environment, with the temperature set at 25 ± 2 °C and humidity maintained at 50 ± 5%, under a 12 h light/dark cycle. The rats were provided with ad libitum access to food and water and were acclimatized to the facility for at least one week before the start of the experiments. The study protocol (9197) was reviewed and approved on 7 March 2025 by the Institutional Animal Care and Use Committee at Texas Southern University, and all procedures were conducted in accordance with ethical guidelines for animal research.

#### 2.4.2. Pharmacokinetic Experimental Design

Diacerein, suspended in an oral vehicle, was administered to the rats at a dosage of 50 mg/kg, with a gavage volume of 0.1 mL per 100 g of body weight. Blood samples, each ranging from 30 to 40 µL, were collected from the tail vein at various time points (0, 0.25, 0.5, 1, 2, 3, 4, 6, 8, and 24 h) following administration. The rats were anesthetized using isoflurane to minimize distress during the blood sampling procedure. Plasma was separated by centrifugation at 8000 rpm for 8 min and stored at −80 °C until further analysis. Plasma samples were processed according to the previously described procedure for calibration, as stated in Section 2.3.1, ensuring consistency in sample preparation.

#### 2.4.3. Data Analysis and Statistical Analysis

Data were analyzed using WinNonlin 3.3 software (Pharsight, Mountain View, CA, USA) with non-compartmental model analysis. Pharmacokinetic parameters, including area under the concentration–time curve (AUC), maximum concentration (C_max_), and terminal half-life (t_1/2_), were calculated for Rhein, Rhein-G1, and Rhein-G2. Results are presented as mean ± standard deviation (SD).

## 3. Results

### 3.1. Method Comparison

Several analytical methods for quantifying Rhein in plasma have previously been published (Table S1). However, most of these methods focus solely on quantifying Rhein itself. The current method, in contrast, enables the simultaneous quantification of Rhein and its major metabolites, Rhein-G1 and -G2. Furthermore, the sensitivity, linear range, and requested sample volume of the current method represent significant improvements over those reported in the literature.

### 3.2. Chromatography and Mass Spectrometry

**Figure 1:**
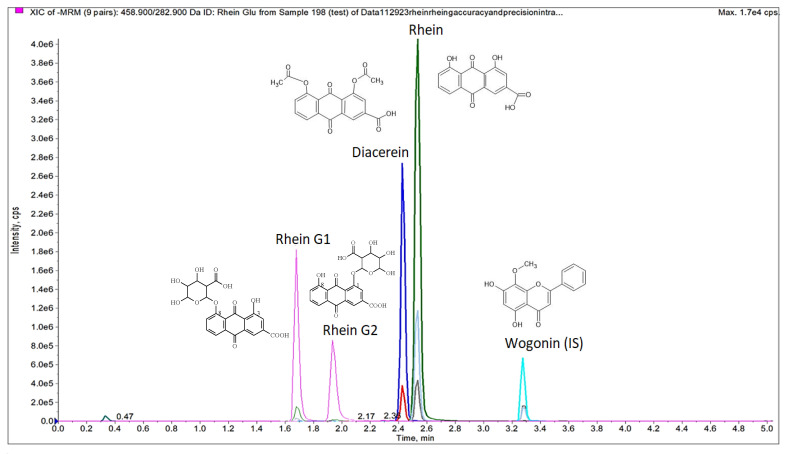
Representative MRM chromatograms of blank plasma with Rhein and its two metabolites.

The method developed in this study was designed to be highly specific, sensitive, and reliable for the simultaneous quantification of Rhein, Rhein-G1, and Rhein-G2 in plasma samples. To the best of our knowledge, this is the first analytical technique that enables the concurrent quantification of these metabolites. A representative chromatogram illustrating the multiple reaction monitoring (MRM) of Rhein and its metabolites spiked in rat plasma is provided in Figure 1. The retention times for diacerein, Rhein, Rhein-G1, Rhein-G2, and the internal standard (wogonin) were 2.4, 2.5, 1.7, 1.9, and 3.3 min, respectively.

For mass spectrometry (MS) detection, both positive and negative ionization modes were initially evaluated by infusing the analytes directly and adjusting compound-specific and instrument-dependent parameters, such as collision energy and declustering potential (Table 1). The results indicated that Rhein, Rhein-G1, and Rhein-G2 exhibited significantly higher sensitivity in the negative ion mode. Consequently, the final method was optimized using the negative ionization mode, enabling the simultaneous detection of all analytes in a single injection, with superior sensitivity and selectivity.

### 3.3. Identification and Quantification of Rhein-G1 and Rhein-G2

The two metabolites were produced through glucuronidation following the protocols published by us previously, suggesting that these two metabolites are Rhein-glucuronides. Additionally, the MS/MS spectra of the two additional peaks were similar to that of Rhein (Figure S1). MS/MS analysis showed a neutral loss of *m*/*z* 176, consistent with glucuronic acid loss [13,14,15]. Rhein glucuronides have been reported previously [17], where two major glucuronide including Rhein-O-8-glucuronide and Rhein-O-1-glucuronide were identified. Acyl-glucuronide may also occur, but it is minor. Additionally, when using the C18 column as the stationary phase and using acetonitrile/water as the mobile phase, Rhein-O-8-glucuronide was elucidated in front of Rhein-O-1-glucuronide [8]. Since we used the same elution system, the metabolites eluted in front were tentatively identified as Rhein-O-8-glucuronide (Rhein-G1) and the other was tentatively identified as Rhein-O-1-glucuronide (Rhein-G2). Further evidence, such as NMR, could be used to confirm the structures of these two metabolites.

The concentrations of Rhein-G1 and Rhein-G2 were quantified using a standard curve for Rhein in UPLC-UV analysis. It should be noted that quantifying Rhein glucuronides using Rhein’s standard curve may introduce minor deviations. However, based on previous observations and published data, a conversion factor between 0.85 and 1.20-fold was considered in HPLC-UV analysis [18]. This range is within acceptable analytical error limits. Accordingly, we proceeded with quantifying Rhein-G1 and Rhein-G2 in the stock solution and prepared a working solution for LC-MS analysis.

### 3.4. Method Validation

#### 3.4.1. Linearity and Lower Limit of Quantification (LLOQ)

The standard curves for Rhein, Rhein-G1, and Rhein-G2 were linear across a range of 7.81 nM to 2000.00 nM (R > 0.99) in plasma samples (Table 2). Accuracy and precision met FDA guidelines, with accuracy between 80.1% and 104.2% and intra-day and inter-day variations below 9.14%. The LLOQ for Rhein and its glucuronides was 7.81 nM.

#### 3.4.2. Accuracy and Precision

Six replicates of QC samples at four concentration levels were analyzed to assess accuracy and precision, with results falling within acceptable ranges (Table 3).

#### 3.4.3. Matrix Effect and Stability

The matrix effect and stability were evaluated using QC samples at three different concentration levels. The matrix effect was found to be within acceptable limits, with relative peak areas comparable to those spiked into water (Table 4).

*Stability*. Stability was assessed after 6 h at 25 °C, 60 days at −80 °C, and after three freeze–thaw cycles. Recovery rates ranged from 81.30% to 104.76%, indicating that stability was within acceptable limits (Table 5).

### 3.5. Application in Pharmacokinetic Studies in Rats

**Figure 2:**
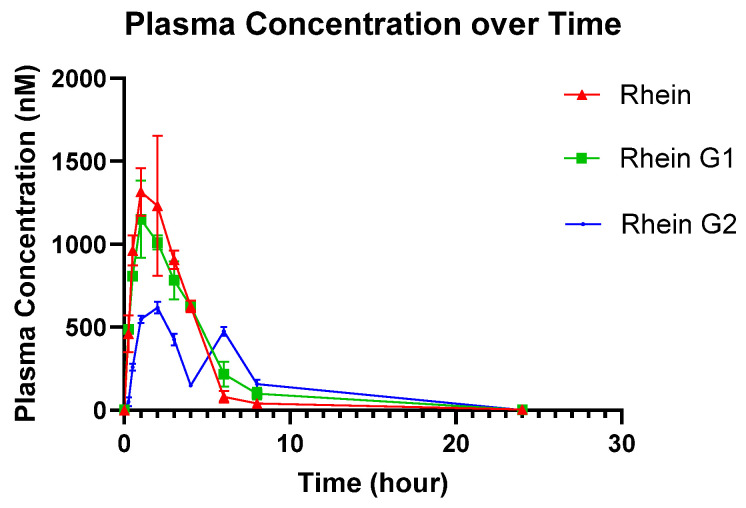
Plasma concentration versus time profiles of the Rhein, Rhein-G1, and Rhein-G2 after oral administration of diacerein (50 mg/kg). Each symbol represents the mean and the error bars indicate the S.D. (*n* = 5).

The mean of the log of plasma concentration–time profiles of Rhein, Rhein-G1, and Rhein-G2 following oral administration are depicted in Figure 2, and the pharmacokinetic (PK) parameters are listed in Table 6. The results indicate that Rhein concentrations in plasma decline rapidly, with a terminal half-life (t_1/2_) of 2.34 ± 1.81 h, which is consistent with human data. The AUC_0-t_ and C_max_ values for Rhein were 4280.15 ± 1576.81 h·ng/mL and 1623.25 ± 334.06 ng/mL, respectively. The PK parameters for Rhein-G1 were comparable to those of Rhein, with t_1/2_, AUC_0-t_, and C_max_ values of 2.78 ± 0.39 h, 3849.045 ± 1983.15 h·ng/mL, and 1351.70 ± 574.46 ng/mL, respectively. In contrast, Rhein-G2 exhibited a longer half-life (t_1/2_: 6.37 ± 2.90 h), with AUC_0-t_ and C_max_ values of 16,491.15 ± 25,430.71 h·ng/mL and 927.56 ± 911.41 ng/mL, respectively.

The data indicate that Rhein achieves a higher maximum concentration (C_max_) in the blood compared to Rhein-G2, but is similar to Rhein-G1. The half-life (T_1/2_) of Rhein-G1 closely resembles that of Rhein, while Rhein-G2 has a somewhat longer half-life. The area under the curve (AUC_0-t_), representing overall drug exposure, is higher for Rhein-G2 than for Rhein. This suggests that, although Rhein reaches higher concentrations initially, Rhein-G2 remains in the system longer, leading to greater overall exposure.

## 4. Discussion

We successfully developed and validated a robust LC-MS method to quantify both Rhein and its two major metabolites. In contrast to the methods published previously (Supplementary Table S1), the major innovation of this method is the ability to quantify both Rhein and Rhein glucuronides (i.e., Rhein-G1, -2) simultaneously. It is necessary to quantify both Rhein and its glucuronides because Rhein undergoes extensive glucuronidation in vivo, as shown in our PK studies (Figure 2) as well as clinical PK studies in humans [7]. Additionally, the glucuronidation of Rhein may enable this compound to undergo enterohepatic recycling facilitated by biliary secretion and microbial hydrolysis, which may significantly impact its efficacy and toxicity. Therefore, the current method is more robust for Rhein in vivo disposition studies. Diacerein, the pro-drug of Rhein, is readily hydrolyzed by carboxylesterases in the GI tract and the liver [19]. This is probably why we did not detect diacerein in the PK study (Figure 2).

Furthermore, the current method requires only 20 µL of plasma with one step of sample preparation, whereas other methods [20,21,22,23,24,25] used from 50 to 100 μL of plasma (Table 1). This reduced plasma volume is particularly advantageous for studies involving limited sample sizes, such as those conducted with small animals. Additionally, the total running time is only 5 min in this method, enabling high throughput for large sample numbers. PK studies indicated that the pro-drug form diacerein was not detected in the plasma, suggesting a rapid release of the active form Rhein from the pro-drug form after oral administration.

We developed this method to quantify Rhein and its major metabolites. The PK application in rats demonstrated that this method could be applied in in vivo studies focusing on Rhein’s disposition by dosing the pro-drug diacerein. However, only one dose of diacerein and one administration route (i.e., oral) were tested. More studies could be conducted focusing on multiple doses and more administration routes (e.g., i.v.), enterohepatic recycling, and drug bio-distribution studies to further elucidate the disposition of Rhein in vivo.

## 5. Conclusions

This study presents the first validated analytical method to simultaneously quantify Rhein and its major metabolites, Rhein-G1 and Rhein-G2, in plasma. The method is sensitive and robust, suitable for pharmacokinetic and phase II metabolism studies. This method can also be applied to human clinical studies, offering even higher sensitivity by utilizing larger blood volumes.

## Figures and Tables

**Table 1:** Compound-dependent parameters for Rhein and its two conjugates in the MRM mode of UHPLC-MS/MS analysis.

Compound	Q1	Q3	DP	CE	CXP
Diacerein	366.8	323.2	−70.0	−20.0	−9.0
Rhein	282.9	238.5	−65.0	−19.0	−9.0
Rhein-G1	458.9	282.9	−70.0	−22.0	−14.0
Rhein-G2	458.9	282.9	−140.0	−22.0	−11.0
IS (wogonin)	283.0	162.0	−80.0	−40.0	−15.0

DP: declustering potential; CE: collision energy; CXP, collision cell exit; IS, internal standard.

**Table 2:** Linear range and LLOQ for Rhein and its two conjugates in MRM mode of UHPLC-MS/MS analysis.

Analyte	Concentration (nM)	LLOQ (nM)
Rhein	2000.00 - 7.81	7.81
Rhein-G1	2000.00 - 7.81	7.81
Rhein-G2	2000.00 - 7.81	7.81

**Table 3:** Precision and accuracy of Rhein and its two conjugates in LC-MS analysis.

Analyte	QC Samples (nM)	Intra-Day (*n* = 6)		Inter-Day (*n* = 18)	
		Precision (RSD, %)	Accuracy (%)	Precision (RSD, %)	Accuracy (%)
Rhein	7.81	8.83	96.90	7.86	98.70
	15.62	5.81	92.00	5.77	97.91
	500.00	3.93	102.26	3.53	102.0
	1000.00	4.82	89.60	3.25	88.20
Rhein-G1	7.81	4.29	100.13	8.08	101.73
	15.62	6.24	97.19	9.14	99.40
	500.00	4.29	102.83	5.11	103.51
	1000.00	3.76	96.21	4.45	94.22
Rhein-G2	7.81	5.45	96.20	6.48	80.1
	15.62	4.32	97.51	3.40	97.90
	500.00	7.83	103.0	4.22	104.2
	1000.00	2.54	93.12	5.21	98.23

**Table 4:** Extraction recovery (%) and matrix effect for Rhein and its two conjugates (*n* = 3).

Analyte	Conc (nM)	Extraction Recovery	Matrix Effect	Matrix Effect
		Mean (%)	SD	Average ± SD (%)
Rhein	7.81	103.27	5.40	83.07 ± 1.67
	15.62	101.20	8.30	101.20 ± 3.36
	500.00	82.31	6.00	97.13 ± 2.58
	1000.00	90.78	12.60	87.24 ± 2.54
Rhein-G1	7.81	103.20	10.54	88.75 ± 3.31
	15.62	86.41	7.50	102.94 ± 2.09
	500.00	91.40	9.05	95.03 ± 5.00
	1000.00	98.40	3.89	94.34 ± 0.61
Rhein-G2	7.81	93.20	4.54	98.75 ± 1.09
	15.62	96.43	6.50	92.94 ± 1.79
	500.00	91.40	5.05	85.07 ± 1.18
	1000.00	88.40	2.89	84.30 ± 0.69

**Table 5:** Stability of Rhein and its metabolites in rat plasma under different storage conditions (*n* = 3).

Analyte	Conc (nM)	25 °C for 6 h		Freeze Thaw		Long Term	
		Stability (%)	CV (%)	Stability (%)	CV (%)	Stability (%)	CV (%)
Rhein	7.81	99.74	10.37	95.35	6.89	93.10	3.20
	15.62	99.08	6.99	102.20	7.33	98.06	6.40
	500.00	99.40	1.75	102.10	6.18	92.12	8.20
	1000.00	97.32	2.97	96.21	1.72	96.30	4.80
Rhein-G1	7.81	104.60	7.22	104.76	6.44	83.19	4.54
	15.62	97.70	6.37	106.91	5.69	85.60	5.57
	500.00	104.62	6.89	97.59	6.29	81.20	6.82
	1000.00	97.94	1.29	97.87	2.61	95.30	4.55
Rhein-G2	7.81	86.40	3.43	89.40	4.20	89.23	8.39
	15.62	81.30	4.23	91.20	5.20	88.34	6.24
	500.00	88.40	5.32	89.30	5.10	81.40	7.12
	1000.00	97.31	1.86	97.75	1.94	95.75	1.98

**Table 6:** Pharmacokinetic parameters of Rhein and its two conjugates after oral administration of Rhein (50 mg/kg) to rat (*n* = 5).

Parameters	Rhein	Rhein-G1	Rhein-G2
T_max_ (h)	1.00 ± 0.14	2.30 ± 1.30	1.80 ± 0.84
C_max_ (nmol/L)	1623.25 ± 334.06	1351.70 ± 574.46	800.74 ± 1007.24
AUC_0~t_ (h·nmol/L)	4280.15 ± 1576.81	3849.045 ± 1983.15	2210.80 ± 2386.71
MRT (h)	2.63 ± 0.35	3.76 ± 0.60	4.91 ± 1.77
T_1/2_ (h)	2.34 ± 1.81	2.78 ± 0.39	5.95 ± 2.98
CL (L/h/kg)	144.9 ± 85.69	2.01 ± 1.29	4.082 ± 1.73
Vz (L/kg)	47.03 ± 33.44	43.08 ± 21.98	36.04 ± 18.2

## Data Availability

All the data are stored in the Research Infrastructure Core (RIC) under the Center for Biomedical and Minority Health Research (CBMHR) at TSU. Data are available for research purposes upon request.

## Supplementary Materials

The following supporting information can be downloaded at: https://www.mdpi.com/article/10.3390/metabo15060407/s1, Figure S1: The MS/MS Spectra of Rhein-G1 (A) and Rhein-G2 (B). The spectra showed a loss of *m*/*z* 176 in MS/MS for both Rhein-G1 and -G2, suggesting a loss of glucuronic acid unit; Table S1: Comparison of this method with those published previously.
