# Supplementary material for: An LC-MS Method to Quantify Rhein and Its Metabolites in Plasma: Application to a Pharmacokinetic Study in Rats
Source: Metabolites. 2025 Jun 17;15(6):407. doi: 10.3390/metabo15060407 (PMC12195357; doi:10.3390/metabo15060407)
Supplement: Supplementary file 1 [file metabolites-15-00407-s001.zip › metabolites-3663393-supplementary.pdf]

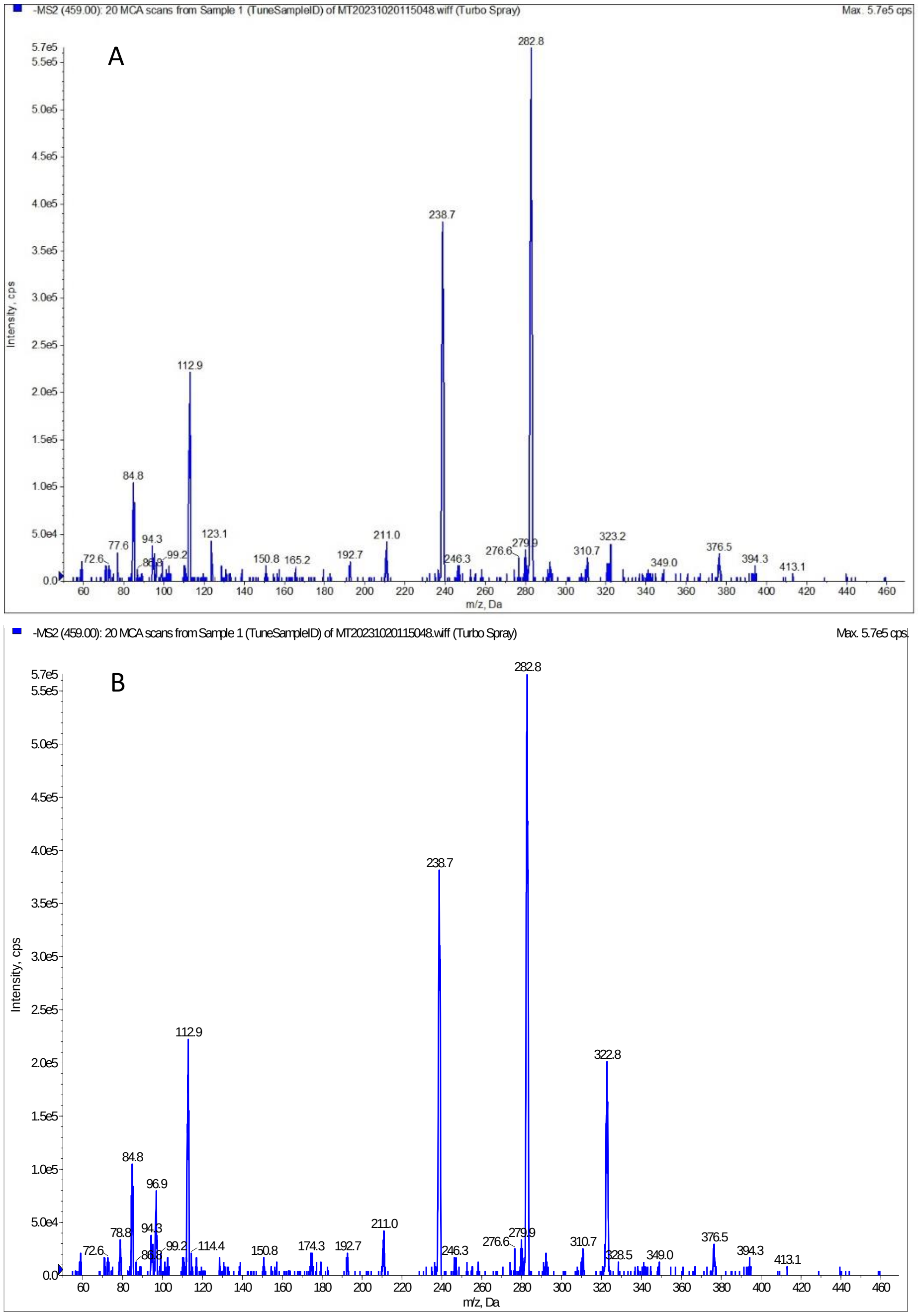

Supplemental Figure S1. The MS/MS Spectra of Rhein-G1 (A) and Rhein-G2 (B). The spectra showed a loss of m/z 176 in MS/MS for both Rhein-G1 and -G2, suggesting a loss of glucuronic acid unit.

Supplemental Table S1. Comparison of this method with those published previously.

No.	Analytes	Sample type	Volume	Extraction	Total run time	Linear range (ng/mL)	Ref
1	Rhein	Human Plasma	10 µL	protein precipitation	12 min	1.0-8,000.0	[20]
2	Rhein	Rat Plasma	50 µL	Protein precipitation	7 min	10.0 -2,000	[21]
3	Rhein	Rat Plasma	60 µL	Protein precipitation	10 min	50.0-2,500	[22]
4	rhein, rhein-8-O-glucoside	Rat Plasma	50 µL	Protein precipitation	10 min	1.0-1,000	[23]
5	Rhein	Rat Plasma	100 µL	Protein precipitation	7.5 min	20.0-800	[24]
6	Rhein	Rat Plasma	100 µL	Protein precipitation	11.1 min	7.8-2,000	[25]
7	Rhein	Rat Plasma	20 µL	Protein precipitation	2.5 min	1.0-1,000	[11]
8	Rhein	Human Plasma	100 µL	Liquid-liquid	5.0 min	5.0-5,000	[8]
9	Rhein, Rhein-8-O-glucuronide, Rhein-1-O-glucuronide	Rat Plasma	20 µL	Protein precipitation	5 min	7.81-2,000.00 (nM)	Current Method
